# Fistula in Ano

**Published:** 1891-09

**Authors:** 


FISTULA IN ANO.—Fistula in ano may be cured by forcibly dilating the sinus and applying sulphate of copper wrapped in loose cotton and pushed to the bottom of the sinus and allowed to remain and dry by slow process. It excites a healing action on the extremity of the sinus. In this way healthy granulations are built up from the bottom, and by degrees they push the cotton plug out and the whole track of the fistula is obliterated.—*Clinique.—Southern Clinic.*

